# The Correlation Between PD-L1 Expression in Metaplastic Breast Cancer and Clinical-Pathological Features and Prognosis

**DOI:** 10.3390/medicina62040726

**Published:** 2026-04-10

**Authors:** Tugba Toyran, Ertuğrul Bayram, Yasemin Aydınalp Camadan, Berksoy Sahin, Kubilay Dalcı, Yusuf Kemal Arslan, Melek Ergin

**Affiliations:** 1 Department of Pathology, Faculty of Medicine, Cukurova University, 01330 Adana, Turkey; erginm@yahoo.com; 2 Department of Oncology, Faculty of Medicine, Cukurova University, 01330 Adana, Turkey; ertugrulbayram84@gmail.com (E.B.); yaseminaydinalp23@gmail.com (Y.A.C.); berksoys@hotmail.com (B.S.); 3 Department of General Surgery, Faculty of Medicine, Cukurova University, 01330 Adana, Turkey; kdalci@cu.edu.tr; 4 Department of Biostatistics, Faculty of Medicine, Cukurova University, 01330 Adana, Turkey; ykarslan@gmail.com

**Keywords:** metaplastic breast carcinoma, PD-L1 expression, tumor-infiltrating lymphocytes, combined positive score, prognosis, immunotherapy, triple-negative breast cancer

## Abstract

*Background and Objectives*: Metaplastic breast carcinoma (MBC) is a rare, aggressive malignancy that is often resistant to conventional chemotherapy and characterized by a triple-negative phenotype. While immune checkpoint inhibition shows promise, the prognostic significance and distribution of programmed death-ligand 1 (PD-L1) expression within the heterogeneous architecture of MBC remain poorly understood. This study aimed to evaluate PD-L1 expression and the density of tumor-infiltrating lymphocytes (TILs) to clarify their roles in patient stratification and overall survival (OS). *Materials and Methods*: We retrospectively analyzed 48 MBC cases diagnosed between 2010 and 2025. PD-L1 expression was quantified using the Combined Positive Score (CPS) with the 22C3 antibody clone across diverse histological components. The density of stromal TIL density was assessed following internationally standardized guidelines. Clinical outcomes and clinicopathological parameters, including metastasis, lymphovascular invasion (LVI), and histological subtype, were correlated with biomarker status using Kaplan–Meier survival analysis and Cox proportional hazards regression models. *Results*: PD-L1 positivity (CPS ≥1) was identified in 72.9% of cases, one of the highest rates documented in literature. Notably, an inverse relationship was observed with PD-L1-negative tumors, which exhibited significantly higher rates of distant metastasis (46.2% vs. 17.1%; *p* = 0.039). Multivariate analysis confirmed that low density of TILs (HR = 9.66; *p* = 0.016), metastasis (HR = 4.40; *p* = 0.023), and the presence of LVI (HR = 3.84; *p* = 0.047) were strong independent predictors of mortality. While PD-L1 status alone did not directly dictate overall survival, mean overall survival was markedly reduced in the low TILs cohort (32.2 months) compared to the high TILs group (114.2 months). *Conclusions*: The high prevalence of PD-L1 expression supports routine screening for immunotherapy eligibility in MBC. Our findings suggest that PD-L1-negative cases represent a high-risk biological subset driven by alternative immune evasion mechanisms. Integrating TIL density with conventional pathological parameters provides a more robust prognostic framework, enabling personalized therapeutic strategies for this challenging malignancy.

## 1. Introduction

Metaplastic breast carcinoma (MBC) is a rare and highly heterogeneous malignancy, accounting for 0.2% to 1% of all invasive breast cancers [1]. Histologically, these tumors manifest a biphasic pattern. This histological structure is characterized by both epithelial and mesenchymal components, including squamous, adenosquamous, fibromatosis-like, spindle cell, and heterologous mesenchymal differentiation (chondroid, osseous, and matrix-producing elements) [1,2,3]. Although MBC is a rare condition, its clinical significance is increasing due to its aggressive biological behavior, tendency to metastasize early, and resistance to conventional chemotherapy regimens [4,5].

In most series, over 90% of MBC cases exhibit a triple-negative (ER−/PR−/HER2−) receptor phenotype, a profile that inherently restricts targeted therapeutic options [1,6]. Despite the absence of a singular pathognomonic mutation for MBC, genomic data demonstrate that these tumors frequently harbor TP53 mutations (approximately 67%), with PIK3CA alterations manifesting less frequently (approximately 23%) [6]. Retrospective cohort studies demonstrate that the five-year recurrence-free survival rate is 57% and the overall survival rate is 66% [7]. The chemotherapy resistance exhibited by MBC has prompted the investigation of alternative treatment strategies, with a particular focus on immunotherapy-based approaches [8].

The survival of tumor cells is dependent upon their ability to evade the immune system via programmed death-ligand 1 (PD-L1) and programmed death-1 (PD-1) checkpoint pathways. PD-L1, which is expressed by both tumor cells and immune cells within the tumor microenvironment, has been shown to bind to PD-1 on activated T lymphocytes, thereby leading to the suppression of antitumor immune responses [9]. Preventing this pathway from being activated via monoclonal antibodies has been demonstrated to be efficacious in a variety of malignancies, including triple-negative breast cancer (TNBC) [10,11]. The efficacy of combining immune checkpoint inhibitors with chemotherapy for advanced TNBC has been substantiated by studies such as the KEYNOTE-355 and IMpassion130 trials, which utilized PD-L1 expression as the primary predictive biomarker of patient response [12,13].

In view of the triple-negative phenotype and the aggressive clinical course of MBC, PD-L1 expression has become a critical clinical focus. A variety of studies have documented that the PD-L1 positivity rate in this uncommon subtype ranges from 17% to 62%, indicating substantial variability. The variability in positivity rates has been demonstrated to be attributed to the specific tests and scoring methods employed (e.g., the distinction between evaluating tumor cells and immune cells) [14,15,16]. In addition, PD-L1 expression levels in MBC are generally higher than those observed in traditional triple-negative and other breast cancer subtypes [6,17]. However, the morphological heterogeneity of MBC poses significant challenges to PD-L1 assessment, as different histological components within the same tumor can exhibit remarkably inconsistent expression patterns [15,18].

Tumor-infiltrating lymphocytes (TILs) have been shown to be of considerable prognostic importance. In the context of standard breast cancer pathology, higher TIL density has been observed to be associated with increased sensitivity to chemotherapy and improved prognosis, particularly in the triple-negative subset [19,20]. However, the assessment of TILs density in MBC can be challenging. The distribution of TILs in MBC exhibits significant variability across different histological areas. For instance, squamous and spindle cell components frequently contain dense lymphocytic infiltrates, while regions characterized by the presence of cartilage or matrix-producing cells are typically deficient in immune cells [15,21]. Therefore, an evaluation of the relationship between PD-L1 expression and TIL density may facilitate a more comprehensive characterization of the intricate immune architecture of MBC. This, in turn, may assist in identifying patients who stand to benefit most from targeted immunotherapy.

While there is a prevailing notion that PD-L1 expression holds considerable therapeutic potential in the context of MBC, the extant literature offers a paucity of data that methodically examine its correlation with precise clinicopathological parameters and patient survival. The true prognostic value of PD-L1 remains a subject of considerable debate; certain cohort studies have indicated longer survival in PD-L1-positive cases [22], while others have reported conflicting findings, associating PD-L1 positivity with a significantly worse prognosis [7]. Of particular significance is the association between PD-L1 expression and conventional prognostic markers, including tumor size, axillary lymph node involvement, and lymphovascular or perineural invasion, and this association remains to be fully elucidated.

In this study, we evaluated PD-L1 expression in 48 MBC cases using the Combined Positive Score (CPS) methodology from the KEYNOTE trials [12]. We analyzed the correlation between PD-L1 expression and various clinicopathological features, including histological subtype, tumor size, lymph node status, lymphovascular invasion, perineural invasion, and distant metastasis. We also examined the relationship among PD-L1 expression, TILs, and patient survival. Our findings could help identify which MBC patients might benefit most from immune checkpoint inhibitors and shed light on the biological behavior of this rare, aggressive breast cancer subtype.

## 2. Materials and Methods

### 2.1. Study Design and Patient Population

In this retrospective cohort analysis, we evaluated 48 patients diagnosed with MBC. All relevant cases were retrieved from the physical archives and electronic hospital records of the Cukurova University Faculty of Medicine, Department of Pathology, covering the period between 2010 and 2025.

Cases were strictly excluded from the study if they lacked sufficient tumor volume for a proper immunohistochemical workup. Additional exclusion criteria encompassed inadequate tissue fixation, severe staining artifacts, or incomplete clinicopathological data.

We extracted baseline parameters directly from patient files. These parameters included patient age, exact tumor dimensions, axillary node status, the presence of lymphovascular or perineural invasion, distant metastasis, and the specific histological subtype. To correctly classify the tumors, we adhered to the criteria outlined in the 5th edition of the WHO Breast Tumors classification [1]. Meanwhile, we determined the pathological stage by applying the 8th Edition of the American Joint Committee on Cancer (AJCC) TNM staging system.

### 2.2. Histopathological Examination

Two experienced breast pathologists thoroughly re-evaluated all available hematoxylin and eosin (H&E)-stained slides. Following this review, they deliberately selected representative tissue blocks that containing adequate areas of invasive carcinoma for subsequent immunohistochemical assays.

### 2.3. Immunohistochemistry

For the immunohistochemistry steps, staining was performed using an antibody against PD-L1. We specifically used the monoclonal mouse anti-human PD-L1 antibody clone 22C3 (Dako, Carpinteria, CA, USA). All of these staining processes were applied on standard formalin-fixed paraffin-embedded (FFPE) tissue sections. Tonsil tissues were always included as positive controls during each staining run.

### 2.4. Evaluation of PD-L1 Expression

Because MBC has a very marked morphological heterogeneity, we sought to evaluate PD-L1 expression across all the identifiable histologic components. These included squamous, spindle cell, chondroid, osseous, and matrix-producing areas. When a tumor showed heterogeneous staining patterns, we did not limit our assessment to a single component. Instead, our CPS scoring was based on an overall assessment of representative tumor zones. We prioritized areas dominated by the invasive tumor, and minor components comprising less than 10% of the total tumor volume were not evaluated separately.

To evaluate the slides, we used the CPS, which is defined as the number of PD-L1-positive tumor cells, lymphocytes, and macrophages divided by the total number of viable tumor cells, multiplied by 100. If the calculated CPS was ≥1, we considered the case as PD-L1 positive [12].

**Figure 1:**
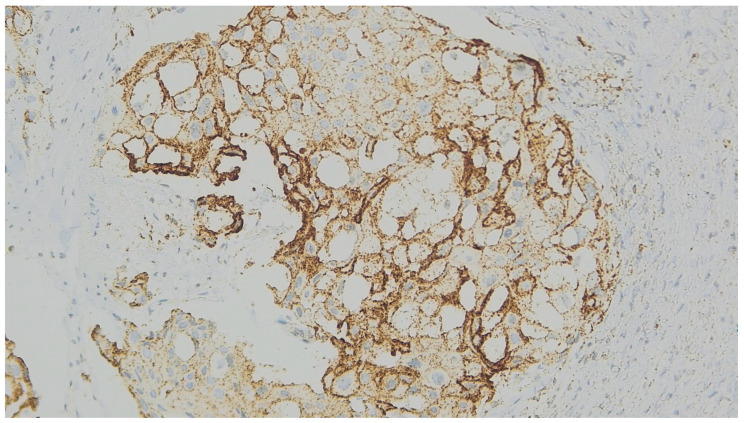
Representative microphotograph of a MBC biopsy specimen with squamous differentiation. There is prominent, strong membranous PD-L1 staining in approximately 60% of the viable tumor cells within the squamous component. (PD-L1 immunohistochemistry, original magnification ×200).

**Figure 2:**
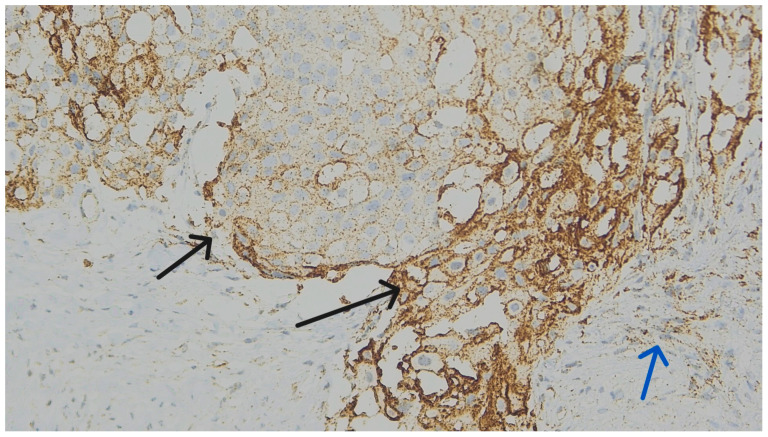
Differential PD-L1 staining at ×200 magnification. Black arrows indicate membranous expression in tumor cells, whereas blue arrows point to cytoplasmic staining in TILs. (PD-L1 IHC).

**Figure 3:**
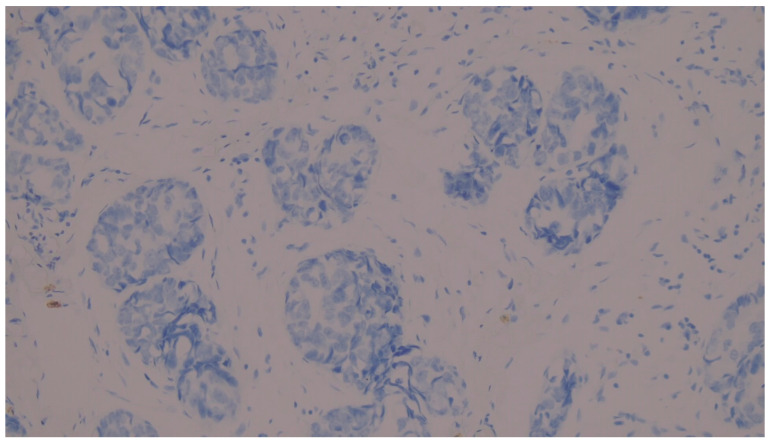
Negative PD-L1 expression in a MBC biopsy specimen. There is a complete absence of PD-L1 immunoreactivity in both the neoplastic cells and the TILs. (PD-L1 IHC, original magnification ×200).

We considered membranous staining in tumor cells (whether partial or complete) and membranous and/or cytoplasmic staining in mononuclear inflammatory cells to be positive (Figure 1, Figure 2 and Figure 3). We strictly excluded any areas with necrosis, crush artifacts, or non-specific background staining from our analysis. Finally, to maintain the reliability of scoring process, at least 100 viable tumor cells had to be present and counted within the evaluated area for a valid CPS assessment.

### 2.5. Evaluation of TILs

We evaluated TILs directly on the available hematoxylin and eosin (H&E)-stained sections. During this assessment, we strictly adhered to the standardized scoring guidelines established by the International Immuno Oncology Biomarker Working Group [19].

We determined stromal TILs by estimating the percentage of the stromal tissue directly occupied by mononuclear inflammatory cells, specifically focusing on the invasive margins of the tumor. Following established international guidelines, we strictly limited our assessment to these stromal TILs. Intratumoral lymphocytes were completely excluded from our final scores.

**Figure 4:**
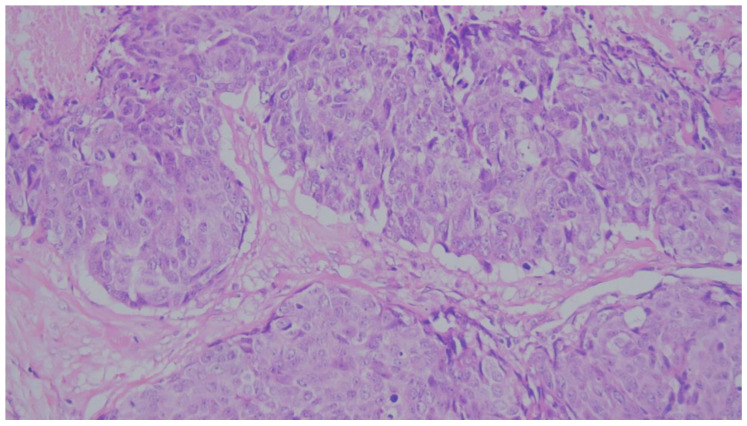
Representative microphotograph of a MBC displaying an “immune-cold” phenotype. There is a complete absence of significant TILs within the tumor nests and the immediate peritumoral stroma. (H&E, original magnification ×200). TILs, categorized as low (<10%).

**Figure 5:**
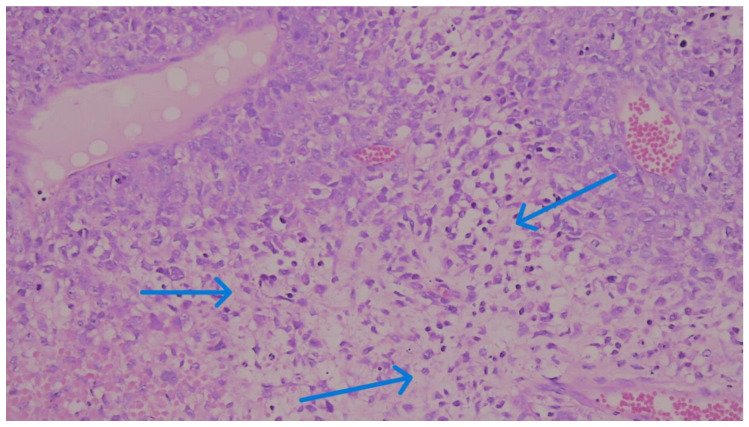
Representative microphotograph of a MBC with intermediate TILs. There is a moderate stromal lymphocytic infiltrate, categorized within the 11–59% range. The inflammatory cells are clearly highlighted by blue arrows within the peritumoral stroma, surrounding the invasive tumor nests. (H&E staining; original magnification ×200).

**Figure 6:**
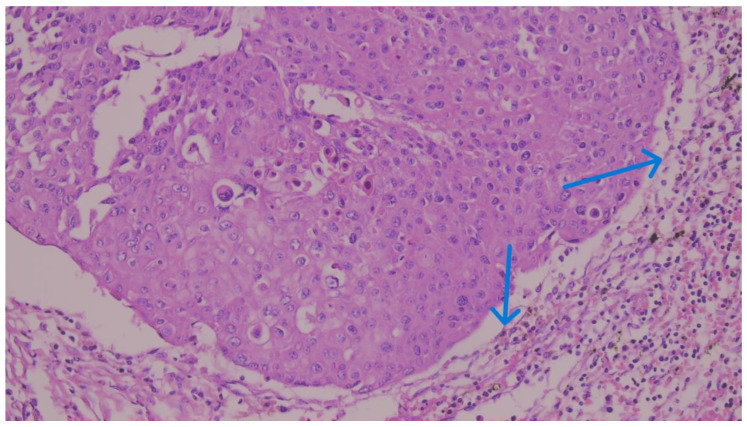
Representative microphotograph of a MBC with high TILs. There is a dense, prominent stromal lymphocytic infiltrate categorized as high (≥60%). The intense inflammatory cell population, highlighting a robust immune microenvironment, is indicated by blue arrows within the peritumoral stroma. (H&E staining; original magnification ×200).

During this evaluation, we avoided regions showing necrosis, unrelated fibrosis, or technical artifacts. To prevent potential sampling bias, we did not just pick isolated “hot spots”; instead, we evaluated the entire invasive tumor footprint (Figure 4, Figure 5 and Figure 6). Based on the final calculated percentages, we stratified all cases into three distinct TIL groups: low (under 10%), intermediate (11% to 59%), and high (60% or greater) [19].

To minimize interobserver bias, two experienced breast pathologists independently evaluated both PD-L1 expression and stromal TILs. Whenever their initial assessments diverged, they reached consensus by jointly reviewing the slides using a multihead microscope.

### 2.6. Statistical Analysis

We presented categorical data as frequencies (*n*) and percentages (%), whereas numerical data were expressed as either the mean ± standard deviation or the median with minimum–maximum ranges. To compare categorical variables across groups, we used the chi-square test. We assessed the normality the distribution of continuous data using the Kolmogorov–Smirnov test. Based on these normality results, we applied the Student’s *t*-test for normally distributed variables and the Mann–Whitney U test for non-parametric data.

For survival analysis, we estimated overall survival (OS) curves using the Kaplan–Meier method and compared group differences with the log-rank test. To identify prognostic factors affecting OS, we built Cox proportional hazards regression models, calculating the relevant hazard ratios and their respective confidence intervals. All statistical analyses were performed using IBM SPSS version 20.0 software (IBM Corp., Armonk, NY, USA). A two-sided *p*-value of less than 0.05 was considered statistically significant for all tests.

## 3. Results

Among the 48 MBC cases analyzed, the mean patient age at diagnosis was 54.4 ± 15.9 years (range: 24–105 years). The median tumor size stood was 4 cm (range: 2–13 cm). Regarding tumor laterality, we noted left breast involvement in 47.9% of the cohort, whereas the remaining cases involved the right breast. We observed multifocal tumor presentation in only two patients (4.2%).

For surgical management, the majority of the patients (66.7%) underwent modified radical mastectomy, whereas segmental mastectomy was preferred for the remainder. When evaluating the receptor profiles, only two cases (4.2%) showed estrogen receptor (ER) positivity, and only one of these was also positive for progesterone receptor (PR). Additionally, HER2 amplification by FISH was confirmed in only two patients.

Among the specific MBC subtypes, the mixed variant was the most common, accounting for 35.4% of our cases. Squamous cell carcinoma accounted for 33.3% of the cohort, closely followed by tumors exhibiting mesenchymal differentiation (31.3%). We have summarized all the comprehensive clinicopathological details of the cohort in Table 1.

Based on our analysis, the distribution of histological subtypes did not differ significantly between the PD-L1-positive and PD-L1-negative cohorts (*p* = 0.439). We noted comparable rates of mixed variant, squamous cell carcinoma, and mesenchymal differentiation across both groups. Likewise, the patient’s PD-L1 status had no apparent impact on whether they presented with low, moderate, or high levels of TILs (*p* = 0.719). We also found significant correlations between PD-L1 expression and other key variables, including pT stage (*p* = 0.655), lymphovascular invasion (*p* = 0.210), lymph node involvement (*p* = 0.431), overall survival status (*p* = 0.498), and the presence of a squamous component (*p* = 0.965). Interestingly, a strong association between PD-L1 positivity and metastatic status was identified (*p* = 0.039). Patients with PD-L1-positive tumors were more likely to remain metastasis-free, whereas distant metastasis occurred at a significantly higher rate in the PD-L1-negative group (Table 2).

Next, we examined how stromal TIL levels were related to the patients’ clinicopathological characteristics and overall prognosis. We detected no significant differences in the distribution of histological subtype among the different TIL categories (*p* = 0.158). TIL density did not show a significant correlation with pT stage (*p* = 0.625), lymphovascular invasion (*p* = 0.099), lymph node positivity (*p* = 0.282), metastatic status (*p* = 0.119), or the presence of a squamous element (*p* = 0.396). However, TIL levels proved to be powerful predictors of mortality (*p* = 0.006). Patients with low TIL density experienced higher mortality, whereas those with moderate-to-high TIL density demonstrated significantly better survival outcomes (Table 3).

During the follow-up period, we recorded 17 patient deaths (35.4%) during the follow-up period. We then mapped various clinicopathological parameters directly against the mortality data, as detailed in Table 4. Interestingly, the specific histological subtype strongly influenced survival (*p* = 0.017). Specifically, the mixed-cell and squamous-cell variants were concentrated heavily in the deceased cohort, whereas tumors exhibiting mesenchymal differentiation were far more frequent among survivors.

Despite its predictive value in other malignancies, the PD-L1 22C3 CPS category did not predict overall survival in our cohort (*p* = 0.498). On the other hand, the degree of stromal infiltration by TILs emerged as a clear prognostic indicator (*p* = 0.006). Cases with sparse TILs experienced noticeably higher mortality, while cases with moderate-to-high lymphocytic infiltration provided had a distinct survival advantage.

We also confirmed several classical risk factors driving fatal outcomes, including lymphovascular (*p* = 0.011) and perineural invasion (*p* = 0.015), advanced axillary nodal stage (*p* = 0.004), and elevated HER2 expression or FISH positivity (*p* = 0.006 and *p* = 0.038, respectively). Metastatic spread dictated the worst prognosis and was tightly correlated with the highest mortality rates (*p* < 0.001). Conversely, factors such as concurrent ductal carcinoma in situ, absolute tumor size, multifocal growth, primary T stage, tumor laterality, and the specific administration of neoadjuvant or adjuvant therapies (including radiotherapy) did not significantly affect survival probabilities in this specific study group.

**Figure 7:**
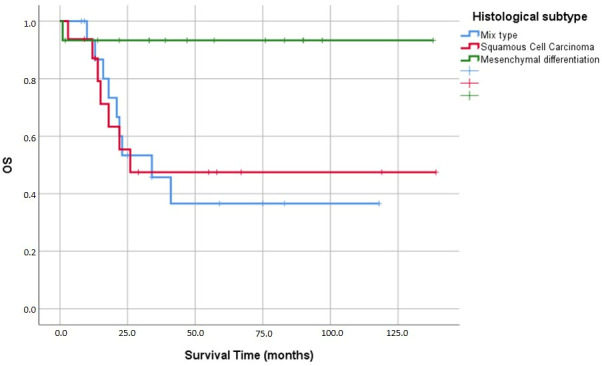
Kaplan–Meier analysis of overall survival (OS) based on histological subtypes. The plot illustrates survival outcomes across different metaplastic breast cancer variants.

In our survival analyses, the mean age of surviving patients stood at 52.1 ± 13.7 years, compared to 58.5 ± 19.0 years for those who died—a difference that was not statistically significant (*p* = 0.185). We did, however, find significant survival disparities among the different histological subtypes (*p* = 0.029). Specifically, mean survival times were 57.7 ± 12.6 months for the mixed variant, 74.5 ± 17.0 months for squamous cell carcinoma, and notably longer at 128.9 ± 8.8 months for tumors demonstrating mesenchymal differentiation (Figure 7).

**Figure 8:**
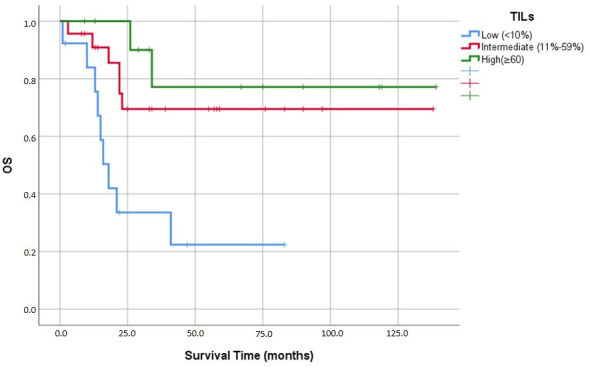
Kaplan–Meier analysis of overall survival (OS) according to TIL levels.

The density of stromal TILs was also a strong prognostic factor (*p* = 0.001). Patients with low TIL levels experienced a markedly shorter mean survival duration (32.2 ± 8.6 months). By contrast, survival extended substantially as lymphocytic infiltration increased, reaching 101.1 ± 12.6 months and 114.2 ± 15.6 months in the moderate and high TILs groups, respectively (Figure 8).

Axillary lymph node burden was inversely correlated with survival times (*p* = 0.021). Node-negative cases enjoyed the longest mean survival (108.2 ± 11.6 months). This duration progressively dropped with increasing nodal involvement: 76.4 ± 14.6 months for 1–3 positive nodes, 35.9 ± 16.5 months for 4–10 nodes, and 35.0 ± 14.1 months for patients with >10 positive nodes.

**Figure 9:**
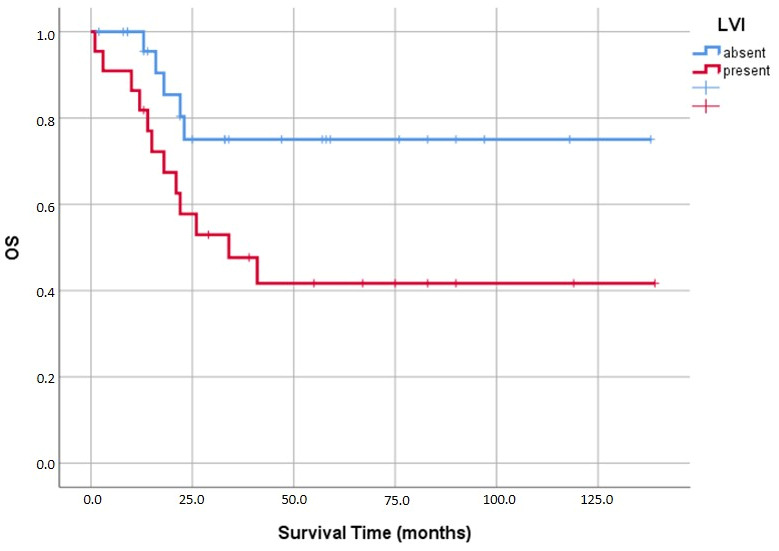
Kaplan–Meier analysis of overall survival (OS) in relation to lymphovascular invasion (LVI).

**Figure 10:**
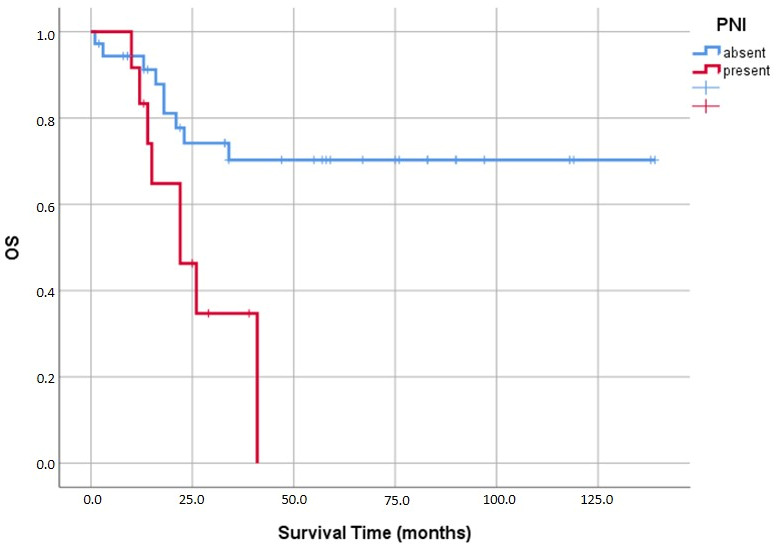
Kaplan–Meier analysis of overall survival (OS) according to perineural invasion (PNI) status.

We observed that both lymphovascular and perineural invasion had similar negative impacts on prognosis. Mean survival plummeted from 108.2 ± 11.6 months in LVI-negative cases down to 69.0 ± 13.3 months when LVI was present (*p* = 0.037) (Figure 9). The effect of PNI was even more drastic (*p* = 0.008); patients without PNI survived an average of 102.8 ± 10.2 months, whereas those with PNI averaged a mere 25.8 ± 3.9 months (Figure 10).

**Figure 11:**
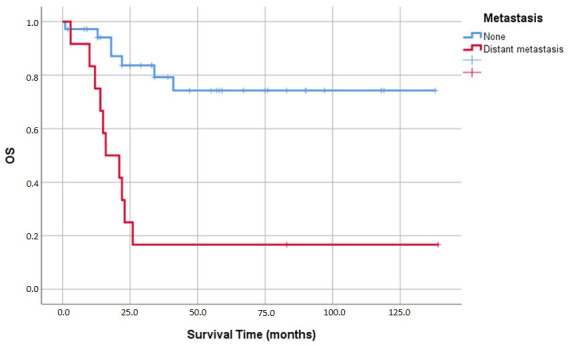
Kaplan–Meier analysis of overall survival (OS) based on the presence of metastasis at diagnosis.

Baseline distant metastasis was associated with the greatest adverse impact on survival (*p* < 0.001). Average survival for patients presenting with metastatic disease was just 36.7 ± 13.3 months, a sharp contrast to the 108.5 ± 9.8 months observed in the non-metastatic cohort (Figure 11).

When we applied Cox proportional hazards regression models, baseline TIL density, lymphovascular invasion, and initial metastatic status emerged as strong, independent predictors of overall survival (Table 5). Relative to the high TILs cohort, patients exhibiting sparse lymphocytic infiltration experienced a substantially increased mortality risk (HR = 9.66; 95% CI: 1.54–60.67; *p* = 0.016). We noted a similarly heightened risk for cases with intermediate levels of TILs (HR = 7.44; 95% CI: 1.17–47.53; *p* = 0.034).

The presence of lymphovascular invasion also severely compromised patient outcomes, independently increasing the likelihood of death (HR = 3.84; 95% CI: 1.02–14.48; *p* = 0.047). As expected, upfront metastatic disease was associated with substantial survival penalty compared with localized cases (HR = 4.40; 95% CI: 1.22–15.81; *p* = 0.023).

Conversely, this regression analysis did not confirm an independent prognostic role for specific histological variants (mixed-type: HR = 4.51; 95% CI: 0.49–41.17; *p* = 0.182; squamous cell carcinoma: HR = 4.64; 95% CI: 0.45–48.02; *p* = 0.198) for the occurrence of perineural invasion (HR = 1.39; 95% CI: 0.46–4.18; *p* = 0.554).

## 4. Discussion

This study examines PD-L1 expression in MBC and how it relates to clinical features and patient outcomes. We found PD-L1 positivity (CPS ≥ 1) in 72.9% of MBC cases, which is notably higher than most previous reports. Interestingly, we observed an unexpected inverse relationship with PD-L1-negative tumors, which actually showed significantly higher rates of distant metastasis. We also found that the density of TILs was a strong independent predictor of overall survival. These findings provide valuable insights into the immune landscape of MBC and may help guide immunotherapy decisions in clinical practice.

### 4.1. PD-L1 Expression Rates in Metaplastic Breast Cancer

We observed PD-L1 positivity (CPS ≥ 1) in 72.9% of our MBC cases, which is among the highest rates reported for this tumor type. This is particularly notable given the wide variation in PD-L1 expression documented across studies, largely attributable to differences in assay platforms, scoring methods, and cutoff values.

Our findings are consistent with studies that assess PD-L1 expression in both tumor cells and immune cells. Voutilainen et al. identified tumor and immune cell positivity in 59% and 62% of MBC cases (*n* = 76), respectively [7]. Grabenstetter et al. reported 95% PD-L1 IC positivity in 42 MBC cases using the SP142 assay with the IMpassion130 criterion (≥1% immune cells). However, applying the stricter CPS ≥ 10 cutoff from KEYNOTE-355 reduced this to 71% [23].

We used the CPS methodology, which accounts for PD-L1 expression on tumor cells, lymphocytes, and macrophages. This approach was validated in the KEYNOTE-355 trial for triple-negative breast cancer [12]. Our CPS ≥ 1 cutoff is more inclusive and captures a broader group of patients who might respond to immunotherapy, consistent with other breast cancer immunotherapy trials [13]. CPS ≥ 1 is the most inclusive and widely reported threshold in the literature for characterizing the prevalence of PD-L1 expression in breast cancer cohorts, and has been used across multiple large studies to describe the immunological landscape of TNBC [24,25].Given the rarity of MBC and the consequently limited sample size in our cohort, the use of CPS ≥ 1 allowed us to maximize the number of evaluable patients and avoid further reduction of already small subgroups.

The high PD-L1 expression we found in MBC is striking compared with that in conventional triple-negative breast cancer. Joneja et al. reported PD-L1 expression in only 9% of TNBC-NOS cases (*n* = 106) versus 46% in of MBC cases (*n* = 75) using the SP142 assay [14]. This suggests that MBC has a more immunogenic microenvironment than that of conventional TNBC, potentially making it a better candidate for immune checkpoint inhibitors.

### 4.2. Correlation with Clinicopathological Features

We found no significant associations between PD-L1 expression and conventional prognostic factors, including histological subtype (*p* = 0.439), stage (*p* = 0.655), lymph node status (*p* = 0.531), lymphovascular invasion (*p* = 0.210), and TIL levels (*p* = 0.719). This suggests that PD-L1 expression in MBC operates independently of traditional pathological parameters and likely reflects tumor-immune interactions rather than tumor burden or invasive potential.

Our lack of correlation between PD-L1 and histological subtype contrasts with some earlier reports. Lien et al. found that squamous components had the highest level of immune cell PD-L1 (66.7%) and stromal TIL (50.0%) levels, whereas matrix-producing and chondroid components showed the lowest level [15]. Voutilainen et al. similarly observed higher PD-L1 and TIL levels in spindle and squamous carcinomas compared with tumors with mesenchymal differentiation [7]. These discrepancies probably reflect differences in how subtypes were classified and scored, especially in cases with mixed morphology.

The independence of PD-L1 from lymphovascular invasion and lymph node status supports the view that immune checkpoint expression mainly reflects host immune response rather than inherent tumor aggressiveness. Clinically, this means that PD-L1 testing might identify immunotherapy-responsive patients regardless of disease stage or risk profile.

### 4.3. The Paradoxical Inverse Relationship: PD-L1 Negativity and Distant Metastasis

One of the most striking findings in our study was the inverse relationship between PD-L1 expression and distant metastasis (*p* = 0.039). We found that 46.2% of PD-L1-negative cases (6/13 patients), developed distant metastasis, compared to only 17.1% of PD-L1-positive cases (6/35 patients). This unexpected pattern challenges conventional assumptions about PD-L1 and disease aggressiveness.

This inverse association suggests that PD-L1-negative MBC may represent a biologically distinct and more aggressive subset that employs immune-evasion mechanisms that do not involve PD-L1 upregulation. Several explanations are possible:

First, PD-L1-negative tumors might employ alternative immune suppression pathways, such as MHC class I loss, immuno-suppressive cytokine secretion (TGF-β and IL-10), or other checkpoint molecules (CTLA-4, LAG-3, and TIM-3) [26,27,28]. These could represent “immune desert” phenotypes with minimal T-cell infiltration and low PD-L1 levels, yet they are still capable of metastasis through non-immune mechanisms.

Second, MBC with mesenchymal or sarcomatoid features may show lower PD-L1 expression while retaining enhanced invasive properties, as EMT programs are known to increase motility, invasiveness, and metastatic capacity independent of immune checkpoint activation [29,30]. Interestingly, mesenchymal differentiation was more common in survivors (45.2% vs 5.9% in deceased patients, *p* = 0.017), suggesting complex relationships between histology, immune phenotype, and outcomes.

Third, PD-L1-positive tumors might be more immunogenic, with higher neoantigen loads driving interferon-gamma-mediated PD-L1 upregulation [31,32]. Despite expressing PD-L1, these tumors may be more susceptible to immune control, limiting metastatic spread. Even a partially suppressed immune response might provide some immunosurveillance.

Finally, PD-L1 expression can change during disease progression. Our assessment used primary tumor specimens, and metastatic sites may differ.

The prognostic significance of PD-L1 in MBC remains debated. Some studies support our findings—Bagbudar et al. found that PD-L1 positivity in immune cells correlated with longer disease-free and disease-specific survival in 85 MBC patients [22], and Chao et al. reported similar associations in 60 cases [33]. However, Voutilainen et al. found that increasing PD-L1 was associated with worse recurrence-free survival (HR 1.08 per 1% increase) and overall survival (HR 1.05 per 1% increase) in 21 patients [7].

In our cohort, PD-L1 expression did not correlate directly with overall survival (*p* = 0.498). Among non-survivors, 77.4% were PD-L1 positive (24/31), compared to 64.7% of survivors (11/17). This lack of direct prognostic impact, combined an inverse association with metastasis, indicates complex relationships between PD-L1 expression, metastatic potential, and survival in MBC.

### 4.4. Clinical Implications of the PD-L1-Metastasis Relationship

The inverse relationship between PD-L1 expression and distant metastasis in our cohort has practical implications for patient management and immunotherapy selection. While PD-L1-positive patients showed lower metastasis rates, their PD-L1 expression indicates active immune–tumor interactions that may responsive to checkpoint blockade. By contrast, PD-L1-negative patients have higher metastatic rates and may need alternative approaches.

For PD-L1-positive metastatic MBC, immune checkpoint inhibitors remain a reasonable option based on their mechanism of action and proven efficacy in triple-negative breast cancer [12,13]. A small case series supports this approach—Kim et al. reported objective responses in 3 of 5 metastatic MBC patients (60%) treated with anti-PD-1 therapy, all of whom had PD-L1-positive tumors [34].

For PD-L1-negative patients (27.1% of our cohort, a 46.2% metastasis rate), alternative strategies should be considered. Dual checkpoint blockade (anti-PD-1/PD-L1 plus anti-CTLA-4) or combinations targeting other immune pathways may overcome PD-L1-independent evasion mechanisms [35]. Adding chemotherapy to immunotherapy can increase tumor immunogenicity through immunogenic cell death, potentially making PD-L1-negative tumors more responsive [36]. Genomic profiling for actionable mutations (PIK3CA, PTEN, and FGFR) may guide targeted therapy use [37]. Given MBC’s rarity and the unique biology of PD-L1-negative cases, clinical trial enrollment is particularly important for these patients.

### 4.5. Prognostic Significance of Tumor-Infiltrating Lymphocytes

TIL density emerged as a powerful independent prognostic factor in our study. In multivariate Cox regression, patients with low TIL levels had a markedly increased risk of mortality compared with those with high TIL levels (HR = 9.66; 95% CI: 1.54–60.67; *p* = 0.016). Intermediate TIL levels also showed significantly worse survival than high TIL levels (HR = 7.44; 95% CI: 1.17–47.53; *p* = 0.034).

Mean survival times differed markedly across TIL categories: 32.2 ± 8.6 months for low TILs (<10%), 101.1 ± 12.6 months for intermediate TILs (11–59%), and 114.2 ± 15.6 months for high TILs (≥60%). This roughly 3.5-fold difference in survival between the low and high TIL groups highlights the profound impact of the adaptive immune response on MBC.

These findings align with the established prognostic value of TILs in triple-negative breast cancer and confirm similar patterns in MBC [19,20]. Chao et al. showed that stromal TILs ≥ 50/mm^2^ correlated with longer disease-free survival in 60 MBC patients [33]. Lien et al. found that stromal TIL positivity (high/intermediate) predicted better survival in a multivariate analysis of 82 cases [15].

High TIL density reflects effective antitumor immunity mediated by cytotoxic T lymphocytes that recognize and eliminate tumor cells. Abundant TILs indicate that the tumor is immunogenic and has triggered a robust adaptive immune response [38]. High TILs may also predict a better response to chemotherapy through immunogenic cell death, in which cytotoxic agents activate the immune system and amplify antitumor immunity [36].

### 4.6. Relationship Between PD-L1 Expression and TILs

Although we found no significant correlation between PD-L1 expression and TIL density (*p* = 0.719), these two biomarkers appear to function as independent prognostic factors that reflect distinct aspects of tumor immunobiology. Among PD-L1-negative cases, the distribution of TILs was 30.8% low, 38.5% intermediate, and 30.8% high. PD-L1-positive cases showed similar patterns: 25.7% low TILs, 51.4% intermediate TILs, and 22.9% high TILs. This independence suggests that PD-L1 expression and TILs are partially distinct features of the MBC immune microenvironment.

This lack of correlation contrasts with the adaptive immune resistance model, where interferon-gamma from tumor-infiltrating T cells induces PD-L1 expression, creating a negative feedback loop [31]. In that model, PD-L1 positivity and high TILs should coincide. However, the relationship in MBC appears more complex, with multiple immune phenotypes [32].

This heterogeneity of immune phenotypes highlights the complexity of the MBC microenvironment and suggests that single biomarkers (PD-L1 or TILs alone) may be insufficient for optimal patient selection. Composite biomarkers combining PD-L1, TIL density, and other immune parameters might better predict immunotherapy responses and outcomes.

### 4.7. Lymphovascular Invasion as an Independent Prognostic Factor

Lymphovascular invasion (LVI) emerged as an independent predictor of worse overall survival in multivariate Cox regression (HR = 3.84; 95% CI: 1.02–14.48; *p* = 0.047). LVI-positive patients had significantly shorter mean survival: 69.0 ± 13.3 months versus 108.2 ± 11.6 months in LVI-negative patients (*p* = 0.037).

LVI was present in 45.8% (22/48) of our cohort and was significantly associated with mortality (*p* = 0.011); 70.6% of deceased patients had LVI compared to only 32.3% of survivors. This aligns with LVI’s established role in hematogenous and lymphatic tumor dissemination [39].

Interestingly, LVI did not significantly correlate with PD-L1 expression (*p* = 0.210); however, higher prevalence of LVI was observed among PD-L1-negative cases (61.5%) compared with PD-L1-positive cases (40.0%). This suggests LVI and PD-L1 represent independent biological processes with LVI reflecting intrinsic invasive capacity, and PD-L1 reflecting tumor–immune interactions. This independence indicates that a comprehensive MBC prognostic assessment should include both traditional pathological parameters and immune biomarkers.

### 4.8. Perineural Invasion and Other Prognostic Factors

Perineural invasion (PNI) showed significant association with mortality in univariate analysis (*p* = 0.015) and Kaplan–Meier analysis (*p* = 0.008), with striking survival differences: 102.8 ± 10.2 months in PNI-negative versus 25.8 ± 3.9 months in PNI-positive patients. However, PNI was not an independent prognostic factor in multivariate Cox regression (HR = 1.39; 95% CI: 0.46–4.18; *p* = 0.554).

This loss of significance in multivariate analysis suggests that PNI’s prognostic impact may be mediated by other variables, particularly metastasis status and LVI. PNI was present in 31.3% (15/48) of cases, which is consistent with rates reported in other aggressive breast cancer subtypes.

Histological subtype significantly associated with survival in univariate analysis (*p* = 0.029), with mean survival of 57.7 ± 12.6 months for mixed type, 74.5 ± 17.0 months for squamous cell carcinoma, and 128.9 ± 8.8 months for mesenchymal differentiation. Notably, mesenchymal differentiation was more common in survivors (45.2%) than in deceased patients (5.9%, *p* = 0.017), suggesting this subtype may have more favorable biology despite MBC’s generally aggressive nature.

However, histological subtype was not an independent prognostic factor in multivariate analysis (mixed type: HR = 4.51, *p* = 0.182; squamous: HR = 4.64, *p* = 0.198). This likely reflects our relatively small sample size and the heterogeneity within subtypes, as many MBC cases contain multiple histological components.

Lymph node status was significantly associated with survival (*p* = 0.021), showing a dose–response relationship: 108.2 ± 11.6 months for N0. 76.4 ± 14.6 months for N1, 35.9 ± 16.5 months for N2, and 35.0 ± 14.1 months for N3. This underscores the prognostic importance of regional lymph node metastasis in MBC.

HER2 positivity by FISH was detected in only 2 patients (4.2%), consistent with MBC’s predominantly triple-negative phenotype [6,7]. FISH positivity was significantly associated with mortality in univariate analysis (*p* = 0.038). However, the small number of HER2-positive cases limits definitive conclusions about HER2’s prognostic significance in MBC, and larger studies are needed to clarify the role of HER2-targeted therapy in this rare subset.

### 4.9. Metastasis Status and Overall Survival

Metastasis status was the most significant predictor of survival in this study. Univariate analysis showed a dramatic reduction in survival for patients with distant metastasis (36.7 ± 13.3 months) compared to those without (108.5 ± 9.8 months; *p* < 0.001). Furthermore, multivariate Cox regression confirmed metastasis as an independent prognostic factor, yielding a Hazard Ratio (HR) of 4.40 (95% CI: 1.22–15.81; *p* = 0.023).

In our cohort, 25% (12/48) of patients presented with distant metastasis. The clinical impact was stark: 58.8% of deceased patients had metastatic disease, compared to only 6.5% of survivors (*p* < 0.001). Notably, we observed a higher metastatic rate among PD-L1-negative cases. This concentration suggests that PD-L1-negative patients represent a particularly high-risk subgroup requiring more intensive surveillance and aggressive therapeutic intervention.

### 4.10. Therapeutic Implications for Immunotherapy in MBC

Our findings underscore the potential for immune checkpoint inhibition in MBC. With a 72.9% PD-L1 positivity rate (CPS ≥ 1), most patients in our cohort met the minimum threshold for immunotherapy. However, defining the optimal CPS cutoff remains a clinical challenge.

While the KEYNOTE-355 trial established a CPS ≥ 10 threshold for pembrolizumab in TNBC [12], applying such stringent criteria might limit eligibility. Nevertheless, Grabenstetter et al. reported that 71% of MBC patients met this higher cutoff [23], suggesting broad applicability even under restrictive protocols.

Emerging case reports provide direct evidence of immunotherapy responsiveness in MBC. Adams et al. described a remarkable response to pembrolizumab combined with nab-paclitaxel in a patient with high PD-L1-expressing metaplastic breast cancer [40]. Ladwa et al. and Gul et al. each documented pathologic complete responses following pembrolizumab-based neoadjuvant regimens [41,42]. Al-Awadhi et al. further reported durable complete remission with atezolizumab plus nab-paclitaxel [43]. Collectively, these cases support the feasibility and clinical benefit of immune checkpoint inhibition in MBC, and reinforce the rationale for incorporating pembrolizumab or atezolizumab into treatment regimens for PD-L1-positive metastatic disease [34].

Conversely, the 27.1% of patients who were PD-L1 negative and found to have the highest metastatic risk require alternative management. This high-risk population should be prioritized for clinical trials, combination immunotherapy, or targeted therapies guided by comprehensive genomic profiling.

### 4.11. Technical Challenges and Assay Standardization

Interpreting PD-L1 expression requires careful consideration of the specific assay platform and scoring methodology employed. In our study, we utilized the 22C3 antibody clone with CPS the FDA-approved companion diagnostic for pembrolizumab in breast cancer [12]. However, significant variability exists in the literature due to the use of diverse clones (e.g., SP142, SP263, 28-8, and E1L3n) and scoring metrics (Immune Cell Score [IC], Tumor Proportion Score [TPS], and Combined Positive Score [CPS]), often leading to discordant positivity rates [23].

Comparative analyses, such as those by Grabenstetter et al., highlight this lack of perfect concordance, noting only 62% agreement for CPS across different assays [23]. While the SP142 assay typically yields higher immune cell staining, the 22C3 clone—used in our study—offers a more balanced assessment across both tumor and immune compartments [44]. These technical nuances underscore the necessity for clinicians to interpret results within the context of the specific validated assay and its corresponding clinical trial data.

The inherent morphological heterogeneity of MBC further complicates PD-L1 assessment. Discordant expression patterns can emerge across different histological components within a single tumor [15,26]. To mitigate this, we evaluated PD-L1 across all identifiable histological subtypes and calculated a representative overall CPS. This strategy aligns with current recommendations for comprehensive sampling in heterogeneous malignancies [23].

### 4.12. Study Limitations

While our study provides significant insight, several limitations warrant consideration. First, the retrospective design and the relatively small sample size (*n* = 48) may limit the statistical power of subgroup analyses. However, given that MBC represents less than 1% of all breast cancers, single-center series typically face similar recruitment challenges. Future multi-institutional collaborations will be essential to validate these findings and explore component-specific PD-L1 interactions on a larger scale.

Second, therapeutic heterogeneity over the 15-year study period (2010–2025) may have influenced survival metrics. Notably, none of the patients received immune checkpoint inhibitors, as these were not yet standard of care. Consequently, our survival data reflect the natural history of the disease and its response to conventional treatments, rather than the predictive value of PD-L1 for immunotherapy efficacy.

Third, our study utilized an integrated CPS approach rather than a component-by-component analysis for mixed tumors. While this provides a holistic overview, it may overlook specific associations unique to individual histological subtypes. Future research incorporating digital pathology and spatial transcriptomics could offer more granular insights into the immune microenvironment of distinct MBC elements.

Furthermore, several technical and biological factors remain to be explored:

• Dynamic Expression: PD-L1 and TILs were assessed only in primary specimens. Given that PD-L1 expression can evolve during metastasis or in response to therapy, paired primary-metastatic analyses are needed [45].
• Biomarker Scope: We did not evaluate genomic signatures (e.g., TMB) or alternative checkpoints such as LAG-3, TIM-3, or CTLA-4. Composite biomarkers integrating genomic and proteomic data likely represent the next frontier in patient stratification [46].
• Immune Characterization: Our assessment was limited to the density of TILs. Future studies utilizing multiplex immunohistochemistry or flow cytometry are necessary to characterize specific T-cell subsets (e.g., CD8+ and FoxP3+), thereby providing a more qualitative understanding of the immune response [33].

### 4.13. Future Directions and Research Perspectives

The clinical landscape of MBC remains complex, and our results highlight several critical avenues for future investigation:

#### 4.13.1. Dedicated Prospective Immunotherapy Trials

The high prevalence of PD-L1 expression in our cohort underscores the urgent need for prospective clinical trials specifically for MBC. Future studies should not only evaluate checkpoint inhibitors but also stratify patients by PD-L1 thresholds (CPS ≥ 1 vs. ≥10), TIL density, and histological subtypes to refine patient selection.

#### 4.13.2. Investigating the PD-L1/Metastasis Paradox

The inverse relationship between PD-L1 expression and metastatic potential observed in our study warrants further mechanistic exploration. Research into alternative immune evasion pathways and epithelial–mesenchymal transition (EMT) signatures in PD-L1-negative MBC could uncover novel vulnerabilities in this high-risk population.

#### 4.13.3. Transitioning Toward Composite Biomarker Models

Moving beyond single-marker assessment, the integration of PD-L1, TIL density, tumor mutational burden (TMB), and gene expression signatures into composite predictive models is essential. Such multiparametric approaches could significantly enhance the precision of immunotherapy response predictions.

## 5. Conclusions

Our study establishes that PD-L1 expression, quantified using the CPS methodology, is present in 72.9% of MBC cases, which is among the highest frequencies documented in the current literature. Paradoxically, PD-L1 negativity was associated with significantly higher rates of distant metastasis (46.2% vs. 17.1%, *p* = 0.039). This finding suggests that PD-L1-negative cases may represent a biologically distinct, highly aggressive subset characterized by alternative immune evasion pathways.

We identified TIL density as a robust independent prognostic factor. Low TIL infiltration was associated with a marked reduction in mean survival time (32.2 months vs. 114.2 months for high TIL infiltration; HR = 9.66, *p* = 0.016). Additionally, LVI remained an independent adverse prognostic indicator (HR = 3.84, *p* = 0.047), whereas PD-L1 expression alone did not directly correlate with overall survival (*p* = 0.498).

These results have direct clinical implications. The high prevalence of PD-L1 positivity warrants routine testing for all MBC patients, as a substantial majority may qualify for immune checkpoint inhibitor therapy, particularly in advanced stages. Furthermore, the prognostic power of the integration of TIL density into standard pathological evaluations to refine risk stratification.

The inverse relationship between PD-L1 status and metastasis underscores the complexity of the MBC immune landscape. PD-L1-negative patients, despite their biomarker status, emerge as a high-risk cohort requiring intensive surveillance and novel therapeutic interventions. Since PD-L1 expression remains independent of traditional parameters such as tumor size and nodal status, it serves as a complementary biomarker for personalized management.

Ultimately, prospective trials stratified by PD-L1 and TIL density are essential to confirm the predictive utility of these markers. Developing composite signatures that integrate immune biomarkers with genomic data will be the next step in optimizing outcomes for this challenging malignancy. As we refine our understanding of the MBC microenvironment, this integrated approach promises to enhance both prognostic precision and therapeutic decision-making.

## Figures and Tables

**Table 1:** Clinicopathological and prognostic features of the patients.

		*n*	%
Histological subtype	Mix type	17	35.4
	Squamous Cell Carcinoma	16	33.3
	Mesenchymal differentiation	15	31.3
PD-L1 22C3 CPS category	Negative	13	27.1
	Positive	35	72.9
Tumor-infiltrating lymphocytes (TILs)	Low (<10%)	13	27.1
	Intermediate (11–59%)	23	47.9
	High (≥60)	12	25.0
Ductal carcinoma in situ	Absent	42	87.5
	Present	6	12.5
Pathological category	0–20	4	8.3
	21–50	24	50.0
	>50	20	41.7
T staging	1	2	4.2
	2	26	54.2
	3	13	27.1
	4	7	14.6
Lymph node category	Negative	26	54.2
	1–3	13	27.1
	4–10	7	14.6
	>10	2	4.2
Lymphovascular invasion	Absent	25	52.1
	Present	23	47.9
N staging	Negative	26	54.2
	1	13	27.1
	2	7	14.6
	3	2	4.2
Neoadjuvant therapy	Absent	28	58.3
	Present	20	41.7
Response to neoadjuvant therapy	No response	5	25.0
	Partial response	12	60.0
	Complete response	3	15.0
Surgical lymph node dissection	Absent	15	31.3
	Present	33	68.8
ERBB2 (HER2)	0	37	77.1
	1	2	4.2
	2	7	14.6
	3	2	4.2
Perineural invasion	Absent	36	75.0
	Present	12	25.0
Adjuvant therapy	Absent	18	37.5
	Present	30	62.5
Adjuvant radiotherapy	Absent	33	68.8
	Present	15	31.3
Metastasis	Distant metastasis	12	25.0
	None	36	75.0

**Table 2:** The relationship between PD-L1 positivity and clinical-pathological parameters and prognosis.

		PD-L1		*p*
		Negative	Positive	
		*n* (%)	*n* (%)	
Histological subtype	Mix type	3 (23.1)	14 (40)	0.439
	Squamous Cell Carcinoma	6 (46.2)	10 (28.6)	
	Mesenchymal differentiation	4 (30.8)	11 (31.4)	
Squamous component	Absent	4 (30.8)	11 (31.4)	0.965
	Present	9 (69.2)	24 (68.6)	
TILs	Low (<10%)	4 (30.8)	9 (25.7)	0.719
	Intermediate (11–59%)	5 (38.5)	18 (51.4)	
	High (≥60)	4 (30.8)	8 (22.9)	
pT Stage	1	1 (7.7)	1 (2.9)	0.655
	2	8 (61.5)	18 (51.4)	
	3	2 (15.4)	11 (31.4)	
	4	2 (15.4)	5 (14.3)	
LVI	Absent	5 (38.5)	21 (60)	0.210
	Present	8 (61.5)	14 (40)	
Lymph node count	Negative	5 (38.5)	21 (60)	0.531
	1–3	5 (38.5)	8 (22.9)	
	4–10	2 (15.4)	5 (14.3)	
	>10	1 (7.7)	1 (2.9)	
Metastasis	None	7 (53.8)	29 (82.9)	0.039
	Distant metastasis	6 (46.2)	6 (17.1)	
Status	Alive	7 (53.8)	24 (68.6)	0.498
	Ex	6 (46.2)	11 (31.4)	

**Table 3:** The relationship between TIL levels and clinical-pathological parameters and prognosis.

		TILs			*p*
		Low (<10%)	Intermediate (11%–59%)	High (≥60)	
		*n* (%)	*n* (%)	*n* (%)	
Histological subtype	Mix type	7 (53.8)	7 (30.4)	3 (25.0)	0.158
	Squamous Cell Carcinoma	2 (15.4)	7 (30.4)	7 (58.3)	
	Mesenchymal differentiation	4 (30.8)	9 (39.2)	2 (16.7)	
Squamous component	Absent	4 (30.8)	9 (39.1)	2 (16.7)	0.396
	Present	9 (69.2)	14 (60.9)	10 (83.3)	
pT Stage	1	0 (0.0)	2 (8.7)	0 (0.0)	0.625
	2	7 (53.8)	11 (47.8)	8 (66.6)	
	3	5 (38.5)	6 (26.1)	2 (16.7)	
	4	1 (7.7)	4 (17.4)	2 (16.7)	
LVI	Absent	6 (46.2)	16 (69.6)	4 (33.3)	0.099
	Present	7 (53.8)	7 (30.4)	8 (66.7)	
Lymph node count	Negative	6 (46.1)	16 (69.6)	4 (33.3)	0.282
	1–3	3 (23.1)	4 (17.4)	6 (50)	
	4–10	3 (23.1)	2 (8.7)	2 (16.7)	
	>10	1 (7.7)	1 (4.3)	0 (0.0)	
Metastasis	None	7 (53.8)	19 (82.6)	10 (83.3)	0.119
	Distant metastasis	6 (46.2)	4 (17.4)	2 (16.7)	
Status	Alive	4 (30.8)	17 (73.9)	10 (83.3)	**0.006**
	Ex	9 (69.2)	6 (26.1)	2 (16.7)	

Values in bold indicate statistically significant results (*p *< 0.05).

**Table 4:** Examination of the relationship between mortality and independent variables.

		Non-Survivors	Survivors	*p*
		*n* (%)	*n *(%)	
Histological subtype	Mix type	8 (25.80)	9 (52.90)	**0.017**
	Squamous Cell Carcinoma	9 (29.00)	7 (41.20)	
	Mesenchymal differentiation	14 (45.20)	1 (5.90)	
PD-L1 22C3 CPS category	negative	7 (22.60)	6 (35.30)	0.498
	positive	24 (77.40)	11 (64.70)	
Tumor-Infiltrating Lymphocytes (TILs)	Low (<10%)	4 (12.90)	9 (52.90)	**0.006**
	Intermediate (11–59%)	17 (54.80)	6 (35.30)	
	High (≥60)	10 (32.30)	2 (11.80)	
Ductal carcinoma in situ	absent	27 (87.10)	15 (88.20)	0.909
	present	4 (12.90)	2 (11.80)	
Tumor size	0–20	3 (9.70)	1 (5.90)	0.203
	21–50	18 (58.10)	6 (35.30)	
	>50	10 (32.30)	10 (58.80)	
Multifocal	0	31 (100.00)	15 (88.20)	0.121
	1	0 (0.00)	2 (11.80)	
T staging	1	1 (3.20)	1 (5.90)	0.115
	2	20 (64.50)	6 (35.30)	
	3	8 (25.80)	5 (29.40)	
	4	2 (6.50)	5 (29.40)	
Lymphovascular invasion (LVI)	Absent	21 (67.70)	4 (29.10)	**0.011**
	Present	10 (32.30)	12 (70.60)	
N staging	0	21 (67.7)	5 (29.4)	**0.004**
	1	8 (25.8)	5 (29.4)	
	2	1 (3.2)	6 (35.3)	
	3	1 (3.2)	1 (5.9)	
Neoadjuvant therapy	Absent	18 (58.10)	10 (58.80)	0.959
	Present	13 (41.90)	7 (41.20)	
Response to neoadjuvant therapy	No response	3 (23.10)	2 (28.60)	0.964
	Partial response	8 (61.50)	4 (57.10)	
	Complete response	2 (15.40)	1 (14.30)	
Localization/Tumor location	Left	16 (51.60)	7 (41.20)	0.489
	Right	15 (48.40)	10 (58.80)	
ERBB2 (HER2)	0	27 (87.10)	10 (58.80)	**0.006**
	1	2 (6.50)	0 (0.00)	
	2	2 (6.50)	5 (29.40)	
	3	0 (0.00)	2 (11.80)	
HER2 FISH	Negative	31 (100.00)	15 (88.20)	**0.038**
	Positive	0 (0.00)	2 (11.80)	
Perineural invasion (PNI)	Absent	27 (87.10)	9 (52.90)	**0.015**
	Present	4 (12.90)	8 (47.10)	
Adjuvant therapy	Absent	9 (29.00)	9 (52.90)	0.102
	Present	22 (71.00)	8 (47.10)	
Adjuvant radiotherapy	Absent	23 (74.20)	10 (58.80)	0.272
	Present	8 (25.80)	7 (41.20)	
Metastasis	None	29 (93.50)	7 (41.20)	**<0.001**
	Distant metastasis	2 (6.50)	10 (58.80)	

Values in bold indicate statistically significant results (*p *< 0.05).

**Table 5:** Multivariable Cox regression results for determining overall survival (OS).

		HR	95% CI for HR (Lower-Upper)		*p*
Histological subtype	MD-reference				0.390
	SCC	4.508	0.494	41.170	0.182
	Mixed	4.637	0.448	48.022	0.198
TILs	High-reference				0.046
	Low	9.659	1.538	60.674	0.016
	Intermediate	7.444	1.166	47.531	0.034
LVI		3.841	1.019	14.475	0.047
PNI		1.394	0.464	4.184	0.554
Metastasis		4.397	1.223	15.807	0.023

HR: hazard ratio, CI: confidence interval, SCC: Squamous Cell Carcinoma, MD: Mesenchymal differentiation, TILs: tumor-infiltrating lymphocytes, LVI: lymphovascular invasion PNI: perineural invasion.

## Data Availability

Data supporting the findings of this study are available from the corresponding author upon reasonable request. The data are not publicly available because of ethical and privacy restrictions related to the patient’s confidentiality.

## Abbreviations

The following abbreviations are used in this manuscript:

MBC	Metaplastic breast carcinoma
PD-L1	Programmed death-ligand 1
TILs	Tumor-infiltrating lymphocytes
OS	Overall survival
CPS	Combined positive score
